# Opioid Administration and Reduction of Pediatric Ileocolic Intussusception

**DOI:** 10.1001/jamanetworkopen.2025.33584

**Published:** 2025-09-24

**Authors:** Karina Burke, Itai Shavit, Daniel M. Cohen, Doug MacDowell, Rakesh D. Mistry, Santiago Mintegi, Simon Craig, Damian Roland, Michael R. Miller, Samina Ali, Naveen Poonai, and the Pediatric Emergency Research Networks (PERN) PAINT Study Group

**Affiliations:** 1 Department of Pediatrics, Schulich School of Medicine and Dentistry, Western University, London, Ontario, Canada; 2 Department of Paediatrics, Hadassah Hebrew University Hospitals, Jerusalem, Israel; 3 Department of Pediatrics, Nationwide Children’s Hospital, Columbus, Ohio; 4 Department of Pediatrics, University of Colorado School of Medicine, Aurora; 5 Pediatric Emergency Department, Biocruces Bizkaia Health Research Institute, Hospital Universitario Cruces University of the Basque Country, UPV/EHU, Bilbao, Basque Country, Spain; 6 Department of Paediatrics, Monash University, Clayton, Victoria, Australia; 7 SAPPHIRE Group, Health Sciences, Leicester University, Leicester, United Kingdom; 8 Paediatric Emergency Medicine Leicester Academic (PEMLA) Group, Children’s Emergency Department, Leicester Royal Infirmary, Leicester, United Kingdom; 9 Department of Epidemiology and Biostatistics, Schulich School of Medicine and Dentistry, Western University, London, Ontario, Canada; 10 Women and Children’s Research Institute, University of Alberta, Edmonton, Canada

## Abstract

**Importance:**

Ileocolic intussusception is an important and painful cause of bowel obstruction in children. Its reduction is also painful, but neither sedation nor analgesia is routinely provided, in part due to concerns surrounding gastrointestinal slowing and consequent failed reduction, and while fentanyl is a potent analgesic, little is known about its association with failed reduction.

**Objective:**

To investigate whether fentanyl is associated with failed reduction in children with intussusception.

**Design, Setting, and Participants:**

This was a secondary analysis of a cross-sectional study from 86 pediatric tertiary care centers in 14 countries conducted between January 1, 2017, and December 31, 2019, characterizing the association of opioid analgesia and sedation with intestinal perforation and failed reduction in children. A consecutive sample of patients 4 to 48 months of age with a discharge diagnosis of ileocolic intussusception who underwent an attempted reduction of intussusception was analyzed, excluding repeat presentations of intussusception and records lacking data for the primary outcome.

**Main Outcome and Measures:**

Age, sex, preexisting gastrointestinal anomalies, time to reduction, and use of prereduction opioid medication data were collected. The primary outcome was the percentage of patients with failed reduction of ileocolic intussusception. Bivariate and multivariable analyses were conducted to determine the association between fentanyl and failed reduction. Data were analyzed in February 2025.

**Results:**

In total, 3184 patients (2038 [64.01%] male), with a median (IQR) age of 17 (9-27) months, were included. Fentanyl was administered within 120 minutes of attempted reduction for 116 of 3167 patients (3.66%). Failed reduction occurred in 484 of 3184 patients (15.20%). In the unadjusted analysis, fentanyl was not associated with failed reduction (odds ratio [OR], 0.66 [95% CI, 0.36-1.22]). In the adjusted analysis, preexisting gastrointestinal anomalies (OR, 4.38 [95% CI, 1.50-12.76]), longer triage to reduction time (OR, 1.04 [95% CI, 1.01-1.07]), and younger age (OR, 0.96 [95% CI, 0.95-0.97]) were associated with failed reduction.

**Conclusions and Relevance:**

In this cross-sectional study of pediatric ileocolic intussusception, fentanyl administration prior to attempted reduction was not associated with failed reduction. These results suggest that fentanyl may be considered a safe therapeutic option to manage children’s intussusception and subsequent reduction-related pain.

## Key Points

**Question:**

Is there an association between fentanyl administration and failed intussusception reduction in children?

**Findings:**

In this cross-sectional study of 3184 children with ileocolic intussusception, fentanyl administration was not associated with failed reduction, but preexisting gastrointestinal anomalies, longer triage to reduction time, and younger age were associated.

**Meaning:**

Findings suggest that fentanyl may be considered a safe option to manage children’s intussusception and subsequent reduction-related pain.

## Teaser

This cross-sectional study investigates whether fentanyl administration to manage pain prior to attempted ileocolic intussusception reduction is associated with failed reduction among children 4 to 48 months of age.

## Introduction

Ileocolic intussusception is the most common type of intussusception in infants^1^ and children and is a leading cause of bowel obstruction.^1,2,3^ The mechanism involves a proximal segment of bowel invaginating into a distal segment.^4,5^ Nonoperative management with hydrostatic or pneumatic enema is the treatment standard.^1,2,4,6,7^ Although there is yet to be an objective quantification of children’s pain and distress during reduction of intussusception,^8^ it is widely believed to be painful and distressing based on both adult and pediatric experience with colonoscopy, where the bowel is also distended with gas and sedation is routinely provided.^9^ Several national position statements have strongly emphasized the importance of adequate management of children’s pain and distress during painful procedures.^10,11,12,13,14^ Unfortunately, sedation and analgesia for reduction of intussusception have traditionally been avoided by most US and European radiologists,^15,16,17,18^ in part due to a belief that opioid-related reduced gastrointestinal motility may reduce the rate of success for reduction of intussusception.

In the original global Pain Management and Sedation in Pediatric Ileocolic Intussusception (PAINT) study, members of our team demonstrated that neither analgesia nor sedation was associated with failed reduction or intestinal perforation. Intravenous morphine was provided to nearly 70% of patients.^19^ Intravenous fentanyl was the second most frequently administered analgesic in the PAINT study.^19^ Intranasal and intravenous fentanyl are increasingly being used in children as an alternative to intravenous morphine in acute care settings^20,21,22,23^ due to their wide availability, ease of administration, tolerability, and rapidity of onset. Determining whether fentanyl is associated with failed reduction is crucial to enable clinicians to make evidence-based decisions in providing comfort to children undergoing reduction of intussusception,^24^ particularly in settings in which sedation is not feasible. With a large global sample, our primary objective was to determine whether fentanyl was associated with failed reduction of intussusception in children. Our secondary objectives were to determine whether other opioids and clinical factors were associated with failed reduction of intussusception.

## Methods

### Design

This cross-sectional study followed the Strengthening the Reporting of Observational Studies in Epidemiology (STROBE) reporting guideline.^25^ This was a substudy of a health record review of children 4 to 48 months of age with a discharge diagnosis of ileocolic intussusception presenting to 86 emergency departments in 14 countries (see study group members in Supplement 1) within the Pediatric Emergency Research Networks between January 1, 2017, and December 31, 2019. The original PAINT study focused on determining the prevalence of analgesia and sedation in children with intussusception and their association with perforation and failed reduction. In the current study, we explored fentanyl and morphine administration as separate covariates. The study was approved by each site’s ethics review board, which waived requirements for individual informed consent because of the low risk nature of the study and the inability to contact individual patients.

### Participants

We included patients 4 to 48 months of age with a sonographic diagnosis of ileocolic intussusception who underwent an attempted reduction using either pneumatic or hydrostatic enema. We excluded repeat presentations of intussusception and records lacking data for the primary outcome of failed reduction of intussusception.

### Data Collection

There were no new data collected for the purposes of this substudy. Data were deidentified to remove personal health identifiers before being entered into a study-specific case report form hosted on the Research Electronic Data Capture platform. The data were extracted from health records, anesthetic records, radiology reports, and operative notes consecutively by a research assistant or site co-investigator. One co-investigator (D.M.) reviewed the data for accuracy, and discrepancies were resolved by discussion with the site investigator. Data extracted included (1) age, sex, and clinical characteristics at the index visit; (2) sedation, defined as any pharmacologic agent with anxiolytic, sedative, dissociative, or anesthetic properties that was administered immediately prior to the reduction attempt; (3) receipt of fentanyl, morphine, or other opioid within 120 minutes prior to reduction attempt; (4) pathological lead point; (5) preexisting gastrointestinal anomalies; (6) duration of time from triage to attempted reduction; (7) reduction method (hydrostatic vs pneumatic); and (8) reduction success.

### Outcome

The primary outcome was the percentage of patients with failed reduction of intussusception associated with fentanyl administration. Secondary outcomes included the percentage of failed reduction associated with the use of other opioids as well as various clinical factors.

### Sample Size Considerations

Based on the expected incidence of intussusception (56 in 100 000),^26^ the number of hospital sites, and the risk of failed reduction of 13.1%,^27^ we anticipated our study period would capture sufficient patients to support a multivariable model with up to 32 covariates. This number of covariates was based on the axiom of 10 to 12 covariates per outcome of interest.^28^

### Statistical Analysis

The data are displayed as counts and percentages for categorical variables and means and SDs or medians and IQRs for continuous variables. Bivariate and multivariable analyses were used to explore the association between the following covariates and failed reduction: fentanyl, morphine, and other opioids within 120 minutes prior to attempted reduction, procedural sedation, age, sex, preexisting gastrointestinal anomalies, time from triage to attempted reduction, reduction technique (hydrostatic vs pneumatic), and enema fluid. We included all covariates in the adjusted (multivariable) models if they had an unadjusted (bivariate) association (statistical significance at *P* < .10). The results were also assessed using a backward elimination model approach. Bivariate and multivariable odds ratios (ORs) and 95% CIs were determined from a generalized mixed-effect logistic regression model with failed reduction as the outcome, exposures of interest as fixed effects, and hospital site as a random effect, using a simple variance components covariance structure with a random intercept. A 2-sided *P* value threshold of <.05 was used to reject the null hypothesis of no association with failed reduction. The analysis was conducted in February 2025. Data were analyzed using complete case analysis. The analysis was performed using SPSS, version 29 (IBM SPSS Inc).

## Results

### Patients

There were 3555 records screened and 3203 records reviewed. After we excluded 19 records with incomplete data on failed reduction, 3184 patients were included in the analysis (Figure). The majority of patients were male (2038 [64.01%]; 1146 [35.99%] female). The median (IQR) age was 17 (9-27) months (Table 1). Preexisting gastrointestinal anomalies were present in 32 of 3184 patients (1.01%) (Table 1). Fentanyl was administered to 116 of 3167 patients (3.66%), and morphine was administered to 297 of 3055 patients (9.72%). Procedural sedation was performed for 333 of 3157 patients (10.55%) (eAppendix in Supplement 2).

**Figure.**
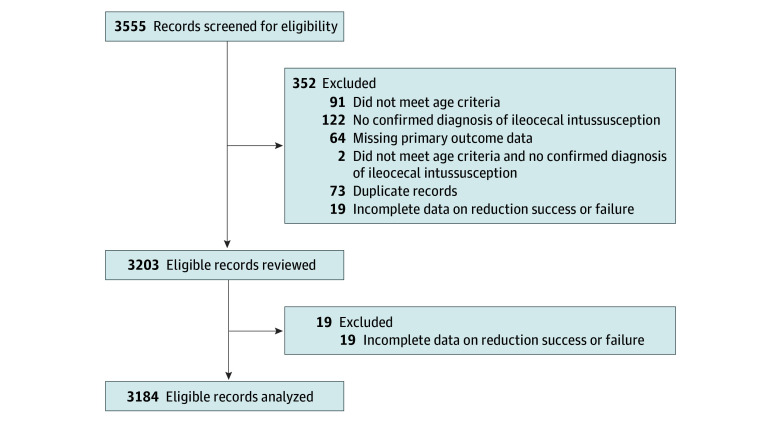
Flow Diagram of Medical Records Reviewed Some records were excluded for more than 1 reason.

**Table 1.** Demographic and Clinical Characteristics

Characteristic	Patients, No. (%)
Age	
No.	2953
Median (IQR), mo	17 (9-27)
Sex	
No.	3184
Female	1146 (35.99)
Male	2038 (64.01)
Clinical presentation	
No.	3184^a^
Abdominal pain	2270 (71.29)
Vomiting	2133 (66.99)
Poor feeding	838 (26.32)
Irritability, fussiness, or episodic crying	837 (26.29)
Bloody stools^b^	782 (24.56)
Diarrhea	718 (22.55)
Lethargy or fatigue	535 (16.80)
Fever	408 (12.81)
Pallor	291 (9.14)
Nausea	177 (5.56)
Constipation	40 (1.26)
Syncope or altered level of consciousness	11 (0.35)
Dehydration or decreased urine output	10 (0.31)
Upper respiratory tract symptoms	23 (0.72)
Abdominal distension	7 (0.22)
No symptoms documented	7 (0.22)
Other symptoms	46 (1.44)
Duration of symptoms prior to ED arrival	
No.	3184
Median (IQR), h	24 (9-48)
Pathologic lead point	
No.	3184
Any^c^	7 (0.22)
Preexisting gastrointestinal anomalies	
No.	3184
Meckel diverticulum	13 (0.41)
Henoch-Schönlein purpura	6 (0.19)
Colonic lymphonodular hyperplasia and ileocecal valve protrusion	2 (0.06)
Umbilical hernia	2 (0.06)
Other anomaly^d^	9 (0.28)

Abbreviation: ED, emergency department.

^a^ Some patients had more than 1 symptom recorded, and there was no documentation of symptoms in 16 patients.

^b^ Stool described as black or red.

^c^ Meckel diverticulum (n = 5), lymph node (n = 1), and terminal ileum duplication cyst (n = 1).

^d^ Each involving 1 patient: Crohn disease; congenital imperforate anus and full repair with colostomy takedown; congenital tracheoesophageal fistula and anorectal malformation; cystic fibrosis; duodenal bulb ulcer and gastrostomy tube; previous intussusception and X-linked lymphoproliferative disorder; previous intussusception, duodenal atresia repair, and malrotation; juvenile polyposis; vertebral defects, anal atresia, cardiac defects, tracheoesophageal fistula, renal anomalies, and limb abnormalities.

### Failed Reduction

There were 484 of 3184 patients (15.20%) with failed reduction who then underwent surgical intervention. Most reductions were attempted using pneumatic enemas (2370 of 3181 [74.50%]). The proceduralist was most commonly a radiologist (3028 of 3184 patients [95.10%]). In the unadjusted analysis, fentanyl used as analgesia or a sedative adjunct was not associated with failed reduction (OR, 0.66 [95% CI, 0.36-1.22]). However, preexisting gastrointestinal anomalies, morphine, other opioids, pneumatic reduction, longer time from triage to reduction, and younger age were associated with failed reduction and were included in the adjusted multivariable model. In the adjusted analysis, only preexisting gastrointestinal anomalies (OR, 4.38 [95% CI, 1.50-12.76]), longer triage to reduction time (OR, 1.04 [95% CI, 1.01-1.07]), and younger age (OR, 0.96 [95% CI, 0.95-0.97]) were associated with failed reduction (Table 2).

**Table 2.** Covariates Associated With Failed Ileocolic Intussusception Reduction

Covariate	No.	Failed reduction, No. (%)		Unadjusted OR (95% CI)^a^	*P* value^b^	Adjusted OR (95% CI)^a^	*P* value^b^
		Yes (n = 484)	No (n = 2700)				
Age, median (IQR), mo	2953	10 (7-20)	18 (9-28)	0.96 (0.95-0.97)	<.001	0.96 (0.95-0.97)	<.001
Sex							
Female (reference group)	1146	161 (14.05)	985 (85.95)	1.15 (0.94-1.41)	.18	NA	NA
Male	2038	323 (15.85)	1715 (84.15)				
Morphine							
Yes	297	63 (21.21)	234 (78.79)	1.62 (1.20-2.18)	.002	1.49 (0.98-2.27)	.06
No (reference group)	2758	394 (14.29)	2364 (85.71)				
Fentanyl							
Yes	112	12 (10.71)	100 (89.29)	0.66 (0.36-1.22)	.18	NA	NA
No (reference group)	3055	468 (15.32)	2587 (84.68)				
Other opioids^c^							
Yes	14	5 (35.71)	9 (64.29)	3.18 (1.06-9.54)	.04	3.70 (0.96-14.26)	.06
No (reference group)	3041	452 (14.86)	2589 (85.14)				
Sedation excluding fentanyl^d^							
Yes	333	53 (15.92)	280 (84.08)	1.07 (0.78-1.46)	.68	NA	NA
No (reference group)	2824	425 (15.05)	2399 (84.95)				
Preexisting gastrointestinal anomalies							
Yes	32	12 (37.50)	20 (62.50)	3.43 (1.66-7.05)	<.001	4.38 (1.50-12.76)	.007
No (reference group)	3146	469 (14.91)	2677 (85.09)				
Time from triage to reduction, median (IQR), h	2459	2.49 (1.28-4.33)	2.32 (1.25-3.82)	1.04 (1.02-1.07)	<.001	1.04 (1.01-1.07)	.003
Reduction method							
Pneumatic	2370	325 (13.71)	2045 (86.29)	1.51 (1.23-1.86)	<.001	0.80 (0.56-1.14)	.21
Hydrostatic (reference group)	811	157 (19.36)	654 (80.64)				
Fluid type							
Water	74	0	74 (100)	NA	>.99	NA	NA
Barium	159	0	159 (100)	NA	>.99	NA	NA
Saline	100	0	100 (100)	NA	>.99	NA	NA
Other	478	157 (32.85)	321 (67.15)	NA	>.99	NA	NA

Abbreviations: NA, not applicable; OR, odds ratio.

^a^ OR and 95% CI compare the odds of failed reduction for each group vs the reference category, an increase in age by 1 month, or an increase in time to reduction by each 1-hour increment.

^b^ *P* values test for a difference between any level of a categorical exposure, or for an OR of 1, for a continuous exposure.

^c^ Opioid drugs are listed in the eAppendix in Supplement 2 and include combinations with nonopioid medications.

^d^ Sedation drugs are listed in the eAppendix in Supplement 2; morphine was not administered during procedural sedation.

## Discussion

The original global multicenter study of intussusception in children (PAINT) found that only a minority of patients (<15%) received analgesia or sedation for reduction.^19^ In the present cross-sectional substudy, we found no association between either fentanyl or morphine and failed reduction. Our results suggest that fentanyl may be considered a safe therapeutic option to manage children’s intussusception and subsequent reduction-related pain. In addition, current practice with morphine administration was considered safe, as we found no increased risk of failed reduction of intussusception associated with morphine administration. However, mounting evidence shows that morphine is associated with adverse effects, such as nausea, vomiting, dizziness,^29,30^ and respiratory depression.^31^ Fentanyl may be a better option than morphine because of greater potency,^31,32,33,34^ fewer adverse effects,^35^ and, specifically, minimal adverse cardiovascular effects.^24^ Moreover, fentanyl can be administered intranasally, making it ideal to quickly and effectively reduce pain in children undergoing reduction of intussusception in settings that lack adequate personnel and resources to sedate children.

In our sample, 15.20% of patients experienced a failed reduction, consistent with the published frequency of 6%-40%.^1,36,37^ Successful nonoperative reduction by hydrostatic or pneumatic enema can obviate the need for surgical intervention and its associated complications for most children.^38^ Our findings largely agree with prior reports in which children under 12 months of age were at increased risk for failed reduction.^1,27,36^ Consistent with prior literature, we also found that longer duration of symptoms was associated with failed reduction.^1,2,3,39,40,41^ The positive association between preexisting gastrointestinal anomalies and failed reduction was described in the original PAINT study.^19^ While prior studies have not described this association, 2 retrospective studies found that a pathological lead point increased the likelihood of failed reduction.^4,5^ The reason for these associations is not entirely clear but suggests the need for tailored approaches to management of intussusception for children at risk of failure of reduction, such as early surgical consultation and specifically for younger children, decreasing the time to reduction using care pathways for expedited diagnostic ultrasound.^36^ The latter has been shown to be beneficial in suspected pediatric appendicitis.^42^ In keeping with the original PAINT study,^19^ neither fentanyl nor morphine administration was associated with failed reduction in this substudy. Fentanyl is arguably a better choice because it is more potent and has a better adverse event profile.^31,32,33,34,35^ Further, it can be given through a variety of routes, including intranasal, which is becoming increasingly popular due to the desire to avoid painful intravenous administration.^43^

Our findings suggest that fentanyl is not associated with failed reduction. These findings are in line with the limited studies that have explored the role of sedation in reduction of intussusception in children. Specifically, esketamine^44^ and midazolam^45,46^ have been found to increase the success rate of hydrostatic reduction, and propofol^17,47^ has been reported to increase the success rate of pneumatic reduction. It has been postulated that the increased intra-abdominal pressure in an agitated child can decrease the progression of the enema medium, thereby increasing the duration of the procedure^17^ and decreasing the success rate.^45^ Fentanyl may exert its beneficial effect by reducing pain and therefore agitation.

In the present sample, sedation was provided to only 10.55% of patients, and the reasons are likely multifactorial. Some centers may have logistic limitations, for example, lacking personnel and equipment to perform procedural sedation in a radiology suite, where reduction typically occurs. Although the reasons were not explored in our analysis, there also may be less comfort with sedating younger children. In addition, there is a lack of guidance regarding sedation for reduction of intussusception. Furthermore, sedating a child for reduction of intussusception remains controversial, despite a lack of evidence indicating that it is unsafe.^48,49,50^ In the United States, only 7% of children with ileocolic intussusception receive sedation during reduction,^15,51^ and there is a lack of support for sedation from most US and European radiologists.^15,16^ This lack of support may relate to a belief that sedation may increase the risk of perforation during reduction^52,53^ and failed reduction.^15^ These notions have been successfully refuted by the results of a systematic review of 849 propofol-based sedations of which 0.6% had intestinal perforation^27^ and of the original PAINT study in which perforation was similarly uncommon (0.4%) and unassociated with sedation.^19^

Only a minority of patients received any opioid within 120 minutes of attempted reduction (13.38%). This finding may reflect a general reluctance to provide analgesia to young children^54^ for painful and distressing procedures, a misconception that analgesia may mask the signs of a surgical condition,^55^ preverbal children’s difficulty with expressing the need for analgesia, or clinician uncertainty with behavioral pain assessment, dosing, and adverse events.^56^ This is in sharp contrast to adults, who receive analgesia for a similar procedure (colonoscopy) that is often performed as a screening test for asymptomatic patients.^13,14^ In the context of our findings, there is now little rationale for withholding fentanyl for children with intussusception.

Both the American Academy of Pediatrics^57^ and Canadian Pediatric Society^10^ have recommended therapeutic approaches to reduce pain and distress in children undergoing diagnostic and therapeutic procedures, although none specifically discuss reduction of intussusception. Intranasal fentanyl has been shown to be equipotent to intravenous morphine for analgesia.^20^ Administration is well tolerated^58^ and can be performed by nurses, with little training required. An intranasal fentanyl protocol has even been shown to decrease time to analgesia administration in children.^22,58^ The American Academy of Pediatrics’ recent clinical practice guideline for opioid prescribing in outpatient settings emphasized that management should involve a multimodal approach including nonpharmacologic strategies and nonopioids, with opioids administered on an as needed basis, and cited that reluctance to prescribe opioids has potentially led to undertreatment of children’s pain.^59^ In settings that lack experience and resources to safely sedate children, fentanyl may be a viable option for reduction of intussusception, but it is critical that clinicians adopt multimodal approaches and develop practice parameters to optimize efficacy while minimizing the risk of adverse events. Future work should urgently focus on how to optimize routine pain assessments in young children using observational scales during clinical presentation and during reduction of intussusception, with a goal to quantify the efficacy of analgesics such as fentanyl.

### Limitations

This study has limitations. Being observational, our study carries the risk of biases by indication in which use of fentanyl and reduction failure are associated with unmeasured confounders. This potential would need to be studied in a prospective randomized clinical trial. Fentanyl has a duration of action of 30 to 60 minutes. We could not be certain that reduction of intussusception fell within this therapeutic window. However, morphine, which has a longer duration of action, was also not associated with failed reduction. This finding suggests that our results would be qualitatively identical if fentanyl was administered within 60 minutes of reduction. We did not explore factors that have previously been associated with failed reduction, such as abdominal pain, fever, tachycardia, hypotension, abdominal distension, and rectal bleeding or currant jelly stools.^1,4,38,60^ This may have reduced the precision of our ORs. Our reasons were that some of these factors, such as abdominal pain and distension could not be objectively verified through health record review. Further, there is inconsistency in the literature regarding the strength of association between failed reduction and factors such as emesis, bloody stools, and abdominal mass.^61^ Ultrasound findings including ascites, trapped fluid, free fluid, and left-sided intussusception have also been associated with increased reduction failure^1,39^; however, these details were not consistently available in radiology reports. Resistive behavior may have been an important factor associated with failed reduction. Unfortunately, these data were not available and their absence emphasizes the importance of quantifying the degree of pain and distress experienced by children during this procedure. The medical specialty overseeing reduction was included in the model because we believed it reflected the difficulty of the case (ie, surgery may oversee more challenging cases). However, the proceduralist’s medical discipline was not captured. Inherent to most medical record reviews, our results were limited by the accuracy and completeness of information contained in the patients’ records. We did not capture data on race, ethnicity, or gender. While we know of no published studies demonstrating race and ethnicity or gender differences in the risk of failed reduction in children, it is plausible that there are race and ethnicity– or gender-related differences in analgesic efficacy. Additionally, given that this was an unfunded global study, 2 data abstractors at each site blinded to the treatments were not possible.

## Conclusions

The findings of this global multicenter cross-sectional study add important new knowledge to the body of evidence characterizing specific factors associated with failed reduction of intussusception in children. Importantly, fentanyl, whether used as analgesia or a sedative adjunct, was not associated with failed reduction, suggesting that it should be considered for primary analgesia and, in settings where sedation may not be feasible, for pain management during reduction of intussusception.

## Notes

Supplement 1.
Pediatric Emergency Research Networks (PERN) PAINT Study Group

Supplement 2. **eAppendix.** Opioids, sedatives, and other proceduralists to accompany Table 2

Supplement 3.
Data Sharing Statement
