# Supplementary material for: Opioid Administration and Reduction of Pediatric Ileocolic Intussusception
Source: JAMA Netw Open. 2025 Sep 24;8(9):e2533584. doi: 10.1001/jamanetworkopen.2025.33584 (PMC12461414; doi:10.1001/jamanetworkopen.2025.33584)
Supplement: Supplement 1. — Pediatric Emergency Research Networks (PERN) PAINT Study Group [file jamanetwopen-e2533584-s001.pdf]

Supplemental Online Content: Nonauthor Collaborators
*First name, last name, and suffix (if applicable) are required and will appear in PubMed.

*Group Name(s): Paediatric Emergency Research Networks (PERN) PAINT Study Group							
***First Name and Middle Initial(s)**	***Last Name**	***Suffix (eg, Jr, III)**	Academic Degrees	Institution	Location (city, state/province, country)	Role or Contribution, eg, chair, principal investigator	Group (if more than 1 Group listed in the byline) and/or Subgroup (eg, Steering Committee)
Nishit	Patel			University of Texas Southwestern Medical Center, Dallas, Texas			PECARN/PEMCRC
Yvette	Wang			Rady Childrens Specialist, Encinitas, California			PECARN/PEMCRC
Alan	Nager			Children's Hospital Los Angeles, Los Angeles, California			PECARN/PEMCRC
Camilo	Gutierrez			Children's National Hospital, Washington, D.C.			PECARN/PEMCRC
Theodore	Heyming			Children's Hospital of Orange County, Orange, California			PECARN/PEMCRC
Rebekah	Burns			Seattle Childrens Hospital, Seattle, Washington			PECARN/PEMCRC
Indi	Trehan			Seattle Childrens Hospital, Seattle, Washington			PECARN/PEMCRC
Emily	Roben			Ann & Robert H. Lurie Children's Hospital of Chicago, Chicago, Illinois			PECARN/PEMCRC
Daniel	Cohen			Nationwide Childrens Hospital, Columbus, Ohio			PECARN/PEMCRC
Doug	MacDowell			Nationwide Childrens Hospital, Columbus, Ohio			PECARN/PEMCRC
Matthew J.	Lipshaw			Cincinnati Children's Hospital Medical Center, Cincinnati, Ohio			PECARN/PEMCRC
Carmen	Sulton			Children’s Healthcare of Atlanta, Atlanta, Georgia			PECARN/PEMCRC
Joyce	Li			Boston Children's Hospital, Boston, Massachusetts			PECARN/PEMCRC
Aderonke	Ojo			Texas Children's Hospital, Houston, Texas			PECARN/PEMCRC
Chris	Pruitt			Medical University of South Carolina			PECARN/PEMCRC
Kimberly S.	Quayle			Washington University School of Medicine, St. Louis, Missouri			PECARN/PEMCRC
Susan M.	Kelly			Nemours/Alfred I. duPont Hospital for Children, New Castle County, Delaware			PECARN/PEMCRC
Alicia	Rolin			University of Michigan, Ann Arbor, Michigan			PECARN/PEMCRC
Shobhit	Jain			Children's Mercy Kansas City, Kansas City, Missouri			PECARN/PEMCRC
Dan	Kornfeld			Children's Mercy Kansas City, Kansas City, Missouri			PECARN/PEMCRC
Justin	Davis			University of Mississippi Medical Center, Jackson, Mississippi			PECARN/PEMCRC
Matthew D.	Thorton			SUNY Upstate Medical University, Syracuse, New York			PECARN/PEMCRC
Kerry	Caperell			Norton Children's Hospital, Louisville, Kentucky			PECARN/PEMCRC
Margaret	Lin-Martore			University of California, San Francisco, California			PECARN/PEMCRC
Iluonose	Amoni			University of Minnesota Masonic Children's Hospital, Minneapolis, Minnesota			PECARN/PEMCRC
Anna	Abrams			Children's Hospital Colorado Anschutz Medical Campus, Aurora, Colorado			PECARN/PEMCRC
Myto	Duong			SIU School of Medicine, Springfield, Illinois			PECARN/PEMCRC
Muhammad	Waseem			Lincoln Medical Center, Bronx, New York			PECARN/PEMCRC
Heather	Territo			John R. Oishei Children's Hospital, Buffalo, New York			PECARN/PEMCRC

Supplemental Online Content: Nonauthor Collaborators
*First name, last name, and suffix (if applicable) are required and will appear in PubMed.

***First Name and Middle Initial(s)**	***Last Name**	***Suffix (eg, Jr, III)**	Academic Degrees	Institution	Location (city, state/province, country)	Role or Contribution, eg, chair, principal investigator	Group (if more than 1 Group listed in the byline) and/or Subgroup (eg, Steering Committee)
Matthew	Steimle			Primary Children's Hospital, Salt Lake City, Utah			PECARN/PEMCRC
Irma	Ugalde			UT-Houston Medical Center			PECARN/PEMCRC
Amanda	Bogie			OU Health Sciences Center, Oklahoma City, Oklahoma			PECARN/PEMCRC
Adrienne L.	Davis			The Hospital for Sick Children, Toronto, Ontario			PERC
Jocelyn	Gravel			CHU Sainte-Justine, Montreal, Quebec			PERC
Evelyne	Doyon-Trottier			CHU Sainte-Justine, Montreal, Quebec			PERC
Neta	Bar Am			Alberta Children's Hospital, Calgary, Alberta			PERC
Graham	Thompson			Alberta Children's Hospital, Calgary, Alberta			PERC
Vikram	Sabhaney			British Columbia (BC) Children's Hospital, Vancouver, British Columbia			PERC
Garth	Meckler			British Columbia (BC) Children's Hospital, Vancouver, British Columbia			PERC
Rini	Jain			Children's Hospital of Eastern Ontario, Ottawa, Ontario			PERC
Samina	Ali			Stollery Children's Hospital, Edmonton, Alberta			PERC
Danilo	Buonsenso			Agostino Gemelli University Policlinic, Rome			REPEM
Silvia	Bressan			University of Padova, Padova			REPEM
Tiziana	Zangardi			University of Padova, Padova			REPEM
Giovanna	Villa			Gaslini Children's Hospital, Genova			REPEM
Martina	Giacalone			University Hospital Meyer, Florence			REPEM
Idanna	Sforzi			University Hospital Meyer, Florence			REPEM
Michelle	Seiler			Children's Hospital Zurich - Eleonore Foundation, Zürich			REPEM
Cyril	Sahyoun			Hôpitaux Universitaires de Genève, Geneva			REPEM
Fabrizio	Romano			University Hospital Bern, Bern			REPEM
Zsolt	Bognar			Heim Pál Children's Hospital, Budapest			REPEM
Szofia	Hajosi-Kalcakosz			Heim Pál Children's Hospital, Budapest			REPEM
Eli	Hershman			Rambam Health Care Campus, Haifa			REPEM
Lisa	Amir			Schneider Children's Medical Center, Petah Tikva			REPEM
Said	Hachimi-Idrissi			Ghent University Hospital, Ghent, Belgium			REPEM
Zanda	Pucuka			Children's Clinical University Hospital, Riga			REPEM
Astra	Zviedre			Children's Clinical University Hospital, Riga			REPEM
Emīlija	Zeltiņa			Children's Clinical University Hospital, Riga			REPEM
Jānis	Kolbergs			Children's Clinical University Hospital, Riga			REPEM
Natalie	Phillips			Queensland Children's Hospital, South Brisbane			PREDICT

Supplemental Online Content: Nonauthor Collaborators
*First name, last name, and suffix (if applicable) are required and will appear in PubMed.

*First Name and Middle Initial(s)	*Last Name	*Suffix (eg, Jr, III)	Academic Degrees	Institution	Location (city, state/province, country)	Role or Contribution, eg, chair, principal investigator	Group (if more than 1 Group listed in the byline) and/or Subgroup (eg, Steering Committee)
Simon	Craig			Monash Medical Centre, Clayton			PREDICT
Meredith	Borland			Perth Children's Hospital, Nedlands			PREDICT
Sharon	O’Brien			Perth Children's Hospital, Nedlands			PREDICT
Jeanette	Marchant			Children’s Hospital at Westmead, Sidney			PREDICT
Virginia	Stanton			Children’s Hospital at Westmead, Sidney			PREDICT
Amit	Kochar			Women's and Children's Hospital, North Adelaide			PREDICT
Gaby	Nieva			Women's and Children's Hospital, North Adelaide			PREDICT
Shane	George			Gold Coast University Hospital, Southport			PREDICT
Victoria	Pennington			Sydney Children's Hospital, Randwick, Sidney			PREDICT
Sarah	Sheedy			Bristol Royal Hospital for Children, Bristol			PERUKI
Mark	Lyttle			Bristol Royal Hospital for Children, Bristol			PERUKI
Jen	Browning			Royal Hospital for Sick Children, Edinburgh, Scotland			PERUKI
Steve	Foster			Royal Hospital for Children, Glasgow, Scotland			PERUKI
Anna	McLoughlin			Royal Hospital for Children, Glasgow, Scotland			PERUKI
Stuart	Hartshorn			Birmingham Children's Hospital, Birmingham			PERUKI
Chaman	Urooj			Evelina London Children's Hospital, London			PERUKI
Lucy	Johnston			Evelina London Children's Hospital, London			PERUKI
Emily	Walton			Royal Alexandra Children's Hospital, Brighton			PERUKI
Charlotte	Harper			Royal Alexandra Children's Hospital, Brighton			PERUKI
Liz	Binham			Sheffield Children's Hospital, Sheffield			PERUKI
Deepika	Subrahmanyam Puthucode			Leicester Royal Infirmary, Leicester			PERUKI
Phil	Peacock			John Radcliffe Hospital, Oxford			PERUKI
James	Conroy			Leeds General Infirmary, Leeds			PERUKI
Rafael	Marañon			Hospital Universitario Gregorio Marañon, Madrid			RISeuP/SPERG
Silvia	Garcia			Cruces University Hospital, Bilbao			RISeuP/SPERG
Nuria	Cahís			Consorci Corporació Sanitària Parc Taulí, Sabadell			RISeuP/SPERG
Amaia	Cámara-Otegui			Hospital Donostia, San Sebastián			RISeuP/SPERG
Arantxa	Gomez			Hospital Universitario Joan XXIII., Tarragona			RISeuP/SPERG
Maria J	Carbonero			Hospital Virgen del Rocio, Sevilla			RISeuP/SPERG
Carlos M	Angelats-Romero			Hospital Francesc de Borja, Valencia			RISeuP/SPERG
Adriana	Yock-Corrales			National Children's Hospital, San José			RIDEPLA

Supplemental Online Content: Nonauthor Collaborators
*First name, last name, and suffix (if applicable) are required and will appear in PubMed.

***First Name and Middle Initial(s)**	***Last Name**	***Suffix (eg, Jr, III)**	Academic Degrees	Institution	Location (city, state/province, country)	Role or Contribution, eg, chair, principal investigator	Group (if more than 1 Group listed in the byline) and/or Subgroup (eg, Steering Committee)
Gabriela	Hualde			Hospital Garrahan, Buenos Aires			RIDEPLA
Indi	Trehan			Lao Friends Hospital for Children, Laos			
Fabian	Spigariol			Reseau Hospitalier Neuchateloi, La Chaux-de-Fonds			
Alex	Donas			Children's Hospital - Lucerne Cantonal Hospital, Lucerne			
Cinthia	Gübeli Linné			Children's Hospital of Eastern Switzerland St.Gallen, St. Gallen			
Laura	Dell’Era			Policlinico of Milan, Milan			
Alessia	Rocchi			Policlinico of Milan, Milan			
Alessia	Pedrazzini			Filippo Del Ponte Hospital, Varese			
Giorgio	Cozzi			Hospital Burlo Garofolo, Trieste			
Egidio	Barbi			Hospital Burlo Garofolo, Trieste			
Laura	Baggio			Hospital for Women and Children, Verona			
Giovana	Fauci			Hospital for Women and Children, Verona			
Angela	Mauro			Santobono-Pausilipon Children's Hospital, Naples			
