# Supplementary material for: Opioid Administration and Reduction of Pediatric Ileocolic Intussusception
Source: JAMA Netw Open. 2025 Sep 24;8(9):e2533584. doi: 10.1001/jamanetworkopen.2025.33584 (PMC12461414; doi:10.1001/jamanetworkopen.2025.33584)
Supplement: Supplement 2. — eAppendix. Opioids, sedatives, and other proceduralists to accompany Table 2 [file jamanetwopen-e2533584-s002.pdf]

Supplementary Online Content

Burke K, Shavit I, Cohen DM, et al. Opioid administration and reduction of pediatric ileocolic intussusception. *JAMA Netw Open*. 2025;8(9):e2533584.
doi:10.1001/jamanetworkopen.2025.33584

eAppendix. Opioids, sedatives, and other proceduralists to accompany Table 2

This supplementary material has been provided by the authors to give readers additional information about their work.

eAppendix. **Opioids, sedatives, and other proceduralists to accompany Table 2**

	**No. (%)**
**Analgesic**	**N= 394**
Morphine (alone)	275 (69.8)
Fentanyl (alone)	73 (18.5)
Morphine + Acetaminophen	16 (4.1)
Nalbuphine (alone)	11 (2.8)
Fentanyl + Acetaminophen	7 (1.8)
Fentanyl + Morphine	3 (0.8)
Oxycodone (alone)	1 (0.2)
Morphine + Ibuprofen	1 (0.2)
Morphine + Acetaminophen + Fentanyl	1 (0.2)
Morphine + Acetaminophen + Ibuprofen	1 (0.2)
Fentanyl + Ibuprofen	1 (0.2)
Fentanyl + Metamizole	1 (0.2)
Fentanyl + Acetaminophen + Ibuprofen	1 (0.2)
Nalbuphine + Acetaminophen	1 (0.2)
**Sedative**	**N= 333**
midazolam (alone)	168 (50.5)
midazolam + ketamine	51 (15.3)
unspecified sedative (alone)	41 (12.3)
propofol (alone)	11 (3.3)
midazolam + propofol	9 (2.7)
ketamine (alone)	8 (2.4)
propofol + fentanyl + sevoflurane + mivacron	6 (1.8)
ketamine + propofol	5 (1.5)
propofol + fentanyl + sevoflurane	5 (1.5)
midazolam + fentanyl	4 (1.2)
midazolam + ketamine + propofol	4 (1.2)
midazolam + propofol + fentanyl	4 (1.2)
propofol + fentanyl + sevoflurane + lystenon	3 (0.9)
midazolam + thiopental	2 (0.6)
sevoflurane (alone)	1 (0.3)
midazolam + sevoflurane	1 (0.3)
ketamine + fentanyl	1 (0.3)
propofol + fentanyl	1 (0.3)
propofol + sevoflurane	1 (0.3)
fentanyl + sevoflurane + tracrium	1 (0.3)
propofol + fentanyl + lystenon	1 (0.3)
propofol + fentanyl + mivacron	1 (0.3)
propofol + sevoflurane + succinylcholine	1 (0.3)
ketamine + fentanyl + sevoflurane + mivacron	1 (0.3)
propofol + fentanyl + sevoflurane + tracrium	1 (0.3)
propofol + sevoflurane + mivacron + remifentanil	1 (0.3)

© 2025 Burke K et al. *JAMA Network Open.*

**Other proceduralists**	**N=376**
Anesthesiologist	200 (53.2)
Pediatrician	49 (13.0)
Emergency physician	34 (9.0)
Pediatric emergency physician	107 (28.4)

© 2025 Burke K et al. *JAMA Network Open*.
