# Supplementary material for: Opioid Administration and Reduction of Pediatric Ileocolic Intussusception
Source: JAMA Netw Open. 2025 Sep 24;8(9):e2533584. doi: 10.1001/jamanetworkopen.2025.33584 (PMC12461414; doi:10.1001/jamanetworkopen.2025.33584)
Supplement: Supplement 3. — Data Sharing Statement [file jamanetwopen-e2533584-s003.pdf]

Data Sharing Statement

Burke. Opioid Administration and Reduction of Pediatric Ileocolic Intussusception. *JAMA Netw Open*. Published September 24, 2025. doi:10.1001/jamanetworkopen.2025.33584

Data
Data available: Yes
Data types: Deidentified participant data
How to access data: Data will be provided upon reasonable request to the corresponding author.
When available: With publication

Supporting Documents
Document types: None

Additional Information
Who can access the data: Data will be made available to researchers whose proposed use of the data has been approved.
Types of analyses: Data for the specified purpose of sub-study conduct.
Mechanisms of data availability: Data will be made available after approval of proposal and with a signed data access agreement.
Any additional restrictions: None
